# Early Identification of Endometrial Malignancy in Postmenopausal Women with Asymptomatic Endometrial Thickening: A Novel Explainable Machine Learning Model

**DOI:** 10.7150/ijms.131192

**Published:** 2026-03-25

**Authors:** Ting Ni, Yanhui Meng, Kefan Peng, Chen Xu, Qiong Fan, Shujun Gao, Yuhong Li, Linlin Yang, Yudong Wang

**Affiliations:** 1 Department of Gynecological Oncology, The International Peace Maternity and Child Health Hospital, School of Medicine, Shanghai Jiao Tong University, Shanghai, China.; 2 Center of Uterine Cavity Disease, The International Peace Maternity and Child Health Hospital, School of Medicine, Shanghai Jiao Tong University, Shanghai, China.; 3 Shanghai Municipal Key Clinical Specialty, Shanghai, China.; 4 Shanghai Key Laboratory of Embryo Original Disease, Shanghai, China.

**Keywords:** asymptomatic, endometrial thickening, endometrial lesions, postmenopausal women, machine learning, SHapley Additive exPlanations

## Abstract

**Objective:**

Early screening and management of asymptomatic postmenopausal women with endometrial thickening are essential to optimize diagnosis and treatment outcomes. However, no unified intervention standards or predictive models for high-risk subgroups exist. This study aimed to develop and validate a SHapley Additive exPlanations (SHAP)-based machine learning (ML) model to identify key risk factors for non-benign lesions in this population.

**Methods:**

Enrolled in this retrospective cohort were 1031 asymptomatic postmenopausal women with endometrial thickening (≥ 5 mm) who underwent hysteroscopy at International Peace Maternity and Child Health Hospital from January 1, 2017, to July 31, 2025. This study comprehensively compiled 33 candidate predictors from accessible clinical datasets, covering demographic characteristics, disease attributes, transvaginal ultrasound results, and laboratory data. Least absolute shrinkage and selection operator (LASSO) regression was adopted for feature selection. Eight machine learning methods (Logistic Regression, Random Forest, Gradient Boosting, XGBoost, LightGBM, Naive Bayes, LDA, QDA) were leveraged to construct the model. Outcome interpretation was performed with the SHAP method, and a dynamic online nomogram was created to support clinical practice.

**Results:**

Parity, endometrial thickness (ET), mean platelet volume (MPV), platelet distribution width (PDW), and D-dimer were identified as independent risk factors. An online nomogram built upon these variables facilitated the real-time prediction of endometrial atypical hyperplasia (EAH)/ endometrial carcinoma (EC). Among eight machine learning models, the Gradient Boosting model achieved the superior performance, with an AUC of 0.763 (95% CI: 0.640-0.865), accuracy of 0.791, sensitivity of 0.667, and specificity of 0.805. Visualized interpretation at the individual patient level was achieved using the SHAP force plot.

**Conclusion:**

We proposed a robust and interpretable ML-driven strategy for EAH/EC risk assessment in postmenopausal women with asymptomatic endometrial thickening. The model demonstrated superior predictive performance and feasibility for population-wide screening, serving as an efficient tool for the risk stratification of early endometrial malignancy prior to surgery and thus preventing overtreatment in low-risk individuals.

## 1. Introduction

Endometrial carcinoma (EC) is the fourth most common malignancy in women and the fifth most common cause of cancer death 1, there will be approximately 69,120 new cancer diagnoses and 13,860 cancer deaths of uterine corpus cancer in 2025 2. Since the mid-2000s, its incidence has risen by ~1% annually, and it is one of only a handful of cancers with increasing mortality, with its mortality rate further rising by 1.5% per year between 2013 and 2022 3. It is prevalent among postmenopausal women, with vaginal bleeding as its classic symptom, accounting for 90%. However, around 15% of postmenopausal EC patients present no symptoms, and endometrial thickening is the sole abnormality identified by transvaginal ultrasound 4. Early identification and timely intervention for such patients are of great importance, yet there is still no universal consensus regarding the recognition and clinical management of these individuals.

Alongside the widespread adoption of transvaginal ultrasound, an increase in the detection rate of asymptomatic endometrial thickening among postmenopausal patients has been observed 5. For postmenopausal bleeding (PMB) patients with an endometrial thickness (ET) exceeding 4 or 5 mm, current guidelines advocate for endometrial biopsy - with postmenopausal endometrial thickening defined as a double-layered thickness of ≥ 5 mm - given that the probability of EC occurrence in patients with ET < 5 mm is less than 1% 6. While applying this cutoff for ET in all asymptomatic women, many low-risk individuals will be unnecessarily subjected to invasive procedures, such as hysteroscopy, resulting in suboptimal healthcare resource allocation, avoidable economic expenditure, and heightened risks of intraoperative injuries and postoperative complications 7. For asymptomatic women, however, there is a paucity of diagnostic criteria or predictive models to identify those at high risk of developing cancer for early surgical intervention, thereby preventing overtreatment of individuals with benign pathologies.

Hematological parameters demonstrate distinct diagnostic superiority due to their inherent stability and ready accessibility 8. Chronic inflammation and hypercoagulability in the tumor microenvironment play critical roles in promoting tumor formation and progression 9, 10. Several inflammatory markers - namely the neutrophil-to-lymphocyte ratio (NLR), platelet-lymphocyte ratio (PLR), and monocyte-lymphocyte ratio (MLR) - alongside coagulation parameters (platelet volume index, serum D-dimer, fibrinogen), have been validated as cost-effective yet robust predictors for histological grading in diverse solid tumors 11, 12. Most current research focus on isolated biomarkers (e.g., endometrial thickness detected by ultrasound) through univariate frameworks, neglecting the synergistic interactions with hematological indices (e.g., mean platelet volume, platelet distribution width, D-dimer), which significantly impairs its clinical applicability and reproducibility 13, 14. Therefore, the present study attempts to utilize a comprehensive set of indicators to develop a visualized, web-based interactive predictive framework in asymptomatic patients with endometrial thickening after menopause.

Machine learning (ML) techniques can promote the risk stratification of complex diseases by integrating multimodal data and performing dynamic time-series analysis 15, thus providing deep insights into clinical information 16. Yet, it is rarely applied to predicting the risk of EC in asymptomatic individuals. The least absolute shrinkage and selection operator (LASSO) regression, as a superior method over conventional approaches, enables effective variable selection in high-dimensional datasets and reduces multicollinearity 17. Hence, we utilize LASSO-based ML methods to enhance the accuracy of predictive models. Additionally, although ML has achieved remarkable advancements over the past decade, ML models are frequently regarded as black boxes with poor interpretability 18. So, to improve its transparency, we employ SHapley Additive exPlanations (SHAP). It can provide the quantitative assessment of how each individual descriptor contributes to the trends in the overall predicted outputs for individual samples 19, rendering the model more visualizable and practical.

In this study, demographical, anthropometric, hematological, and ultrasound data were extracted from postmenopausal asymptomatic patients with endometrial thickening before hysteroscopic surgery. The present study integrates eight ML algorithms to establish predictive models to assess the risk of endometrial malignancy. Furthermore, SHAP-based interpretability is designed to elucidate the relative contribution of each feature. Moreover, a simple-to-use web-based tool is constructed. Focused on optimizing personalized management of asymptomatic women, this work seeks to realize early detection of malignant tumors prior to surgery, consequently preventing unnecessary invasive procedures.

## 2. Materials and Methods

### 2.1 Study design and patient selection

This retrospective cohort study initially analyzed 1603 postmenopausal asymptomatic patients with endometrial thickening detected by transvaginal ultrasound who underwent hysteroscopy at the International Peace Maternity and Child Health Hospital (IPMCH) from January 1, 2017, to July 31, 2025. At transvaginal sonography, endometrial thickness was measured at its thickest point from the anterior to the posterior wall in the sagittal plane of the uterus. Calipers were positioned perpendicular to the outer edge of the endometrium 20. The inclusion criteria were as follows: (1) patients with primary amenorrhea for more than 1 year without a pharmacological explanation; (2) no symptoms of abnormal vaginal bleeding or discharge; (3) transvaginal ultrasound showing double-layer endometrial thickness ≥ 5 mm 21; (4) women who underwent hysteroscopic resection with a definitive histopathological diagnosis; (5) having complete clinical data within one week before surgery; and (6) informed consent and voluntary participation in this study. Exclusion standards criteria were as follows: (1) presence of abnormal bleeding or discharge; (2) lacking recent preoperative blood tests or transvaginal ultrasound examinations performed at our institution; (3) intake of certain drugs (such as anti-inflammatory or antiplatelet medications); (4) women with serious diseases such as chronic cardiac insufficiency, liver diseases, and mental diseases; (5) patients who were unable to cooperate or declined to participate in this study. This project was approved by the Institutional Review Board of IPMCH: ref. no. (GKLW) 2022-17).

### 2.2 Data collection

According to clinical significance and data availability, we collected data on 33 potential candidate variables. All data were extracted from the hospital electronic medical record system and subsequently verified by manual review to ensure accuracy and completeness. The following variables were recorded: demographics and clinical characteristics: age, body mass index (BMI), age at menopause, duration of menopause, gravidity, parity, number of miscarriages, hypertension, diabetes, hyperlipidemia; ultrasound findings: preoperative endometrial thickness measured by transvaginal ultrasound and preoperative serum parameters: white blood cells (WBC), platelet count (PLT), neutrophil count, lymphocyte count, monocyte counts, hematocrit (HCT), mean corpuscular volume (MCV), red cell distribution width (RDW), mean platelet volume (MPV), platelet distribution width (PDW), platelet-larger cell ratio (P-LCR), platelet thrombocytocrit (PCT), prothrombin time (PT), international normalized ratio (INR), activated partial thromboplastin time (APTT), fibrinogen, thrombin time (TT), D-dimer, fibrin degradation products (FDP). In addition, inflammatory and coagulation-related composite indices were calculated, including neutrophil-to-lymphocyte ratio (NLR), platelet-to-lymphocyte ratio (PLR), and monocyte-to- lymphocyte ratio (MLR).

### 2.3 Feature Screening

Least Absolute Shrinkage and Selection Operator (LASSO) regression analysis (glmnet 4.1.8 in R) was applied for variable selection and regularization 22. As an extension of generalized linear models, this approach enabled (1) the selection of predictive variables via L1-penalization, and (2) the construction of a parsimonious predictive signature. Continuous variables were standardized (mean = 0, SD = 1), and the optimal λ value was determined using 10-fold cross-validation based on the minimum criterion, which allowed the identification of predictors while minimizing overfitting. Meanwhile, univariate and multivariate analyses were performed simultaneously. Combined with LASSO regression, we selected clinical variables with the highest discriminatory power to construct a predictive nomogram for EAH/EC diagnosis. Furthermore, an interactive web-based dynamic nomogram application was developed using Shiny (version 0.13.2.26) for clinical application 23.

### 2.4 Analysis and evaluation of machine learning models

In parallel, eight machine learning models, including Logistic Regression (LR), random forest (RF), gradient boosting, extreme gradient boosting (XGBoost), LightGBM, Naive Bayes, linear discriminant analysis (LDA), and quadratic discriminant analysis (QDA) were built by using Python (sklearn 0.22.1, xgboost 1.2.1, lightgbm 3.2.1). We then trained and tested the aforementioned models with 10-fold repeated sampling 24, assessed feature importance, and selected the optimal model. The performance of the models was assessed by discrimination, calibration, and clinical utility. Discrimination was determined by the area under the receiver operating characteristic (ROC) curve (AUC), which ranges from 0.5 (no discrimination) to 1 (perfect discrimination) 25. Calibration was visually assessed via calibration plots and quantitatively with the Brier score, comparing the predicted probabilities against the actual observed frequencies of EC progression. Clinical utility was evaluated using decision curve analysis (DCA), which calculated the net benefit across a range of threshold probabilities. Given the anticipated class imbalance, Python (Sklearn 0.22.1) was used to plot Precision-Recall (PR) curves, and the average precision value (AP) with 95% confidence intervals was reported 26. In the PR curve, the x-axis represented recall, defined as the proportion of true positive cases correctly identified by the model, while the y-axis represented precision (positive predictive value), defined as the proportion of predicted positive cases that were truly positive. Models with PR curves positioned closer to the upper-right region indicated better performance. The area under the PR curve, summarized as AP, provided an overall measure of performance, with higher values indicating improved balance between precision and recall. Furthermore, the learning curve was generated to evaluate whether the optimal model would benefit from additional training data and to detect potential overfitting 27.

### 2.4 SHAP interpretability analysis

SHapley Additive exPlanations (SHAP) was employed to interpret the predictions of the model 13. A SHAP value quantified the contribution of an individual feature to a model's prediction for a given observation. It indicates “how much” and “in which direction” a specific variable shifted the model's prediction relative to the baseline. A positive SHAP value indicated that a feature increased the predicted risk, whereas a negative SHAP value indicated a risk-reducing effect. The absolute SHAP value quantified the strength of the feature's contribution to an individual prediction. To examine detailed feature effects and interactions, a SHAP dependence plot was generated for key predictors. It tracked the cumulative effect of feature contributions on the model output from the base probability to the final prediction. Global interpretation was performed using the SHAP summary plot. By calculating SHAP values, this method could quantify the contribution of each feature, intuitively indicating whether the impact was positive or negative 28. Furthermore, SHAP waterfall and force plots were used to illustrate individualized predictions for two representative cases.

### 2.5 Statistical analysis

Normally distributed continuous data are described as mean ± standard deviation (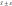), while non-normally distributed data are reported as median (interquartile range, [P25, P75]). Categorical data are expressed as number (%). The Kolmogorov-Smirnov and Levene's tests were used to evaluate data normality and homogeneity of variance, respectively. Consequently, the Wilcoxon test was applied for non-normally distributed continuous data. Categorical variables were compared with the Chi-square or Fisher's exact test. Statistical significance was set as *p* < 0.05. Data analyses were performed using IBM SPSS Statistics Version 26.0 (IBM Corp., Armonk, NY, USA) and R version 4.3.2 (http://www.r-project.org). A web-based dynamic nomogram was constructed with Shiny, version 0.13.2.26, and Python Version 3.8.0 was employed to construct predictive models.

## 3. Results

### 3.1 Patient characteristics

**Figure 1:**
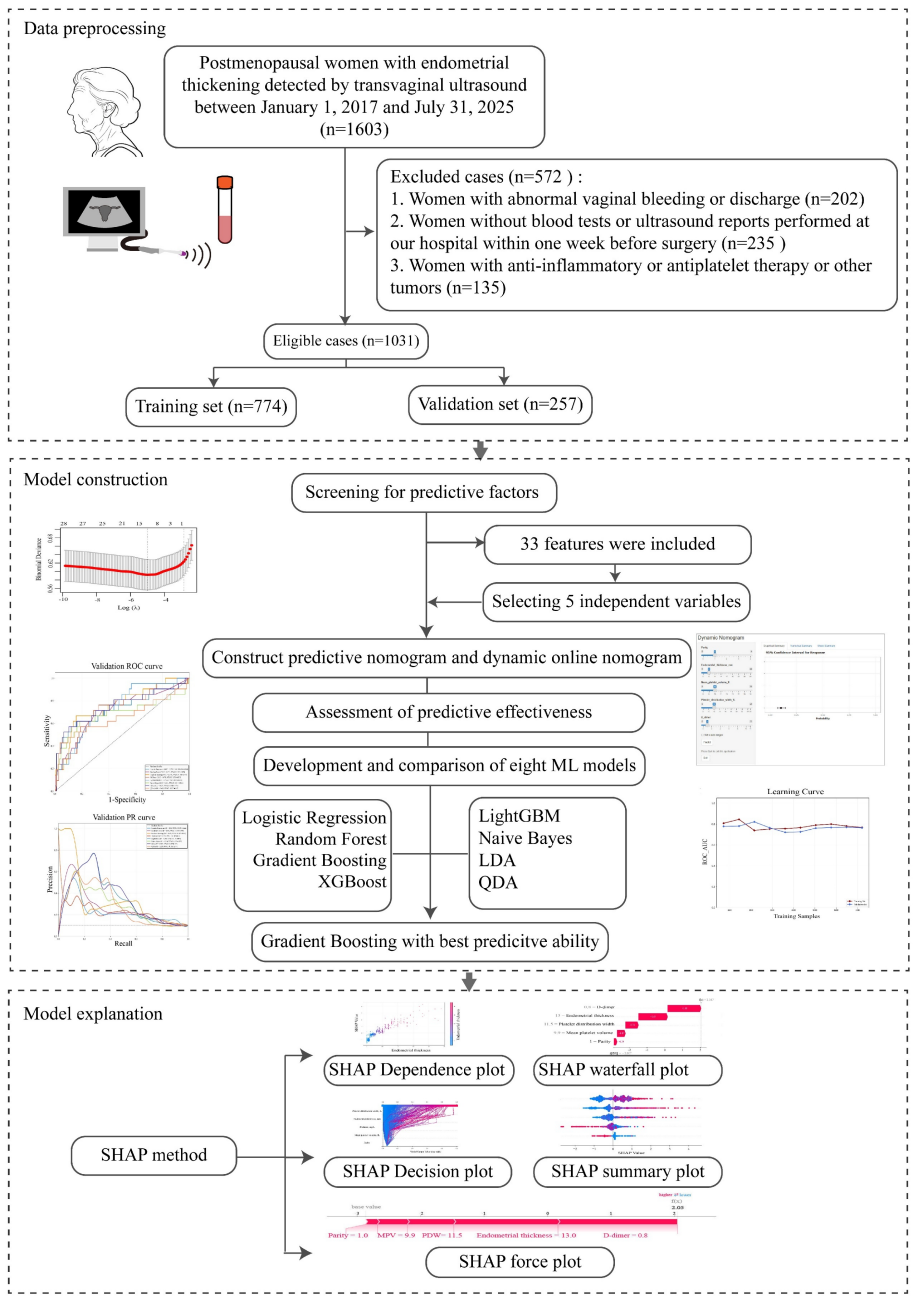
** Flowchart of study design and analytical procedures**.

A total of 1603 postmenopausal women with asymptomatic endometrial thickening were initially collected. Among them, 572 women were excluded because of abnormal vaginal bleeding or discharge (n = 202), missing preoperative blood tests or transvaginal ultrasound examinations at our institution within one week before surgery (n = 235), or current administration of anti-inflammatory or antiplatelet agents and a history of other cancers (n = 135). Finally, 1031 patients were enrolled in this study. A flowchart of patient selection and the study design was presented in Figure 1. Subsequently, the pathological results of hysteroscopic endometrial curettage in these patients were summarized in Table 1, with benign findings accounting for 89.7% of all diagnoses. Endometrial polyps were the most common benign lesion (63.7%), followed by simple hyperplasia (13.8%). Endometrial atypical hyperplasia was detected in 2.9% of patients, and endometrial carcinoma in 7.4%. Among these cases, endometrioid carcinoma was the predominant histological subtype in our study, accounting for 86.84% of the 76 EC cases. The remaining cases included endometrial clear cell carcinoma (3.95%) and serous endometrial carcinoma (3.95%), carcinosarcoma (2.63%), mesonephric-like adenocarcinoma (1.32%), and mucinous endometrial carcinoma (1.32%), as presented in Supplementary Figure 1.

Furthermore, patients were randomly divided into training cohort (n = 774) and validation cohort (n = 257). Stratified sampling was applied to ensure that the class distribution in the training and test sets was highly consistent with that in the original dataset, thereby preserving the model's high sensitivity for the malignant class. Baseline characteristics were comparable between the two cohorts, and no significant differences were detected (*p* > 0.05, Supplementary Table 1). In the training set, patients were stratified into two subgroups based on histopathological diagnosis: 693 patients in the benign endometrial lesions group and 81 in the non-benign (EAH/EC) group (Table 2). Patients with EAH/EC were younger than those with benign lesions (median age 61 vs. 64 years, *p* = 0.017) and had a shorter duration of menopause (*p* = 0.029). Nulliparity was observed at a significantly higher rate in the EAH/EC group compared with the benign lesion group (7.4% vs. 2.6%, *p* = 0.043). In addition, endometrial thickness was markedly greater in patients with EAH/EC than in those with benign lesions (11.37 ± 5.11 mm vs. 7.77 ± 2.89 mm, *p* < 0.001). Regarding laboratory parameters, patients with EAH/EC showed higher levels of RDW, MPV, PDW, P-LCR, and D-dimer (all *p* < 0.05). No significant differences between-group differences were observed in age at menopause, comorbidities (hypertension, diabetes, and hyperlipidemia), gravidity, number of miscarriages, BMI, routine blood cell counts, coagulation parameters other than D-dimer, or inflammatory indices (all *p* > 0.05).

### 3.2 Establishment and evaluation of the nomogram

**Figure 2:**
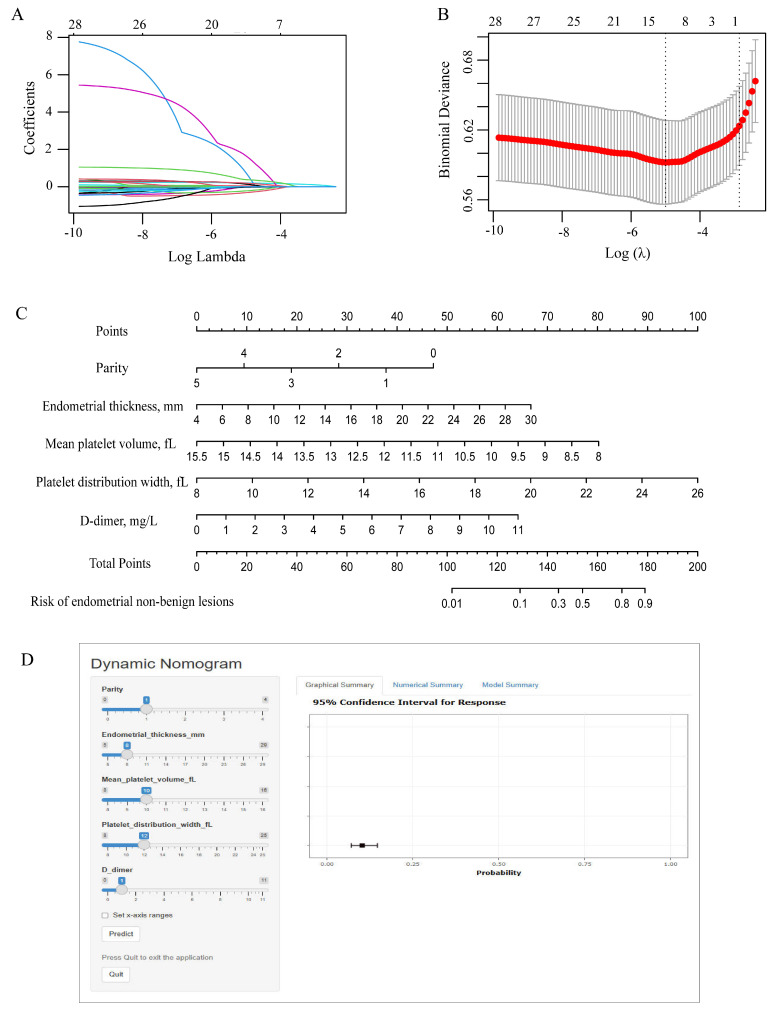
** Feature selection and nomogram construction.** (A) Plots of the LASSO coefficient profiles. Coefficient paths showing variable shrinkage with increasing regularization (λ). (B) Optimization of the regularization parameter (λ): using 10-fold cross-validation. Binomial deviation and log(λ) curves were plotted. (C) Variable ranges and point allocation in the nomogram. (D) Web-based dynamic nomogram for individualized risk prediction accessible at https://yixuelietu.shinyapps.io/Dynamic_Nomogram/. LASSO: Least Absolute Shrinkage and Selection Operator.

To avoid overfitting and select important clinical variables, LASSO regression was used. A total of 33 candidate variables encompassing demographic characteristics, clinical features, transvaginal ultrasound findings, and laboratory data were entered into the LASSO regression model for analysis (Figure 2A-B). Based on the minimum criterion (lambda_min = 0.013), 12 variables with non-zero coefficients were identified, including age, age at menopause, duration of menopause, gravidity, parity, BMI, endometrial thickness, RDW, MPV, PDW, PLR, and D-dimer. The detailed results were presented in Supplementary Figure 2.

Multivariable logistic regression analysis was subsequently performed to further identify independent predictors of EAH/EC (Table 3). Parity (OR = 0.522, 95% CI: 0.314-0.868, *p* = 0.012), endometrial thickness (OR = 1.250, 95% CI: 1.178-1.327, *p* < 0.001), MPV (OR = 0.387, 95% CI: 0.165-0.913, *p* = 0.030), PDW (OR = 1.636, 95% CI: 1.130-2.368, *p* = 0.009), and D-dimer (OR = 1.538, 95% CI: 1.130-2.094, *p* = 0.006) remained significantly associated with EAH/EC. These five variables were ultimately selected for subsequent analysis.

Additionally, a predictive nomogram incorporating these five independent factors was constructed to assess the risk of non-benign endometrial lesions (Figure 2C). The nomogram demonstrated excellent discriminative performance, with an AUC of 0.780 (95% CI: 0.737-0.835) in the entire cohort, 0.784 (95% CI: 0.726-0.842) in the training cohort, and 0.777 (95% CI: 0.690-0.864) in the validation cohort (Supplementary Figure 3A-C). Calibration plots showed good agreement between predicted and observed probabilities in the overall, training, and validation cohorts, indicating satisfactory model calibration (Supplementary Figure 3D-F). Decision curve analysis demonstrated that the nomogram model yielded a higher standardized net benefit than the treat-all and treat-none strategies across a wide range of threshold probabilities in the entire cohort, training cohort, and validation cohort (Supplementary Figure 3G-I). These findings indicated favorable clinical utility of the model for risk-stratified decision-making. For an individual patient, the value of each predictor was first located on the corresponding axis of the nomogram, and the associated score was determined by projecting vertically to the points scale. The scores for all predictors were then summed to generate a total score, which was subsequently mapped to the probability scale to estimate the individualized risk of endometrial non-benign lesions. In addition, a dynamic online nomogram (https://yixuelietu.shinyapps.io/Dynamic_Nomogram/) was further developed to facilitate individualized risk prediction by allowing real-time input of predictor values and providing estimated probabilities with corresponding confidence intervals (Figure 2D).

### 3.3 Model development and validation

**Figure 3:**
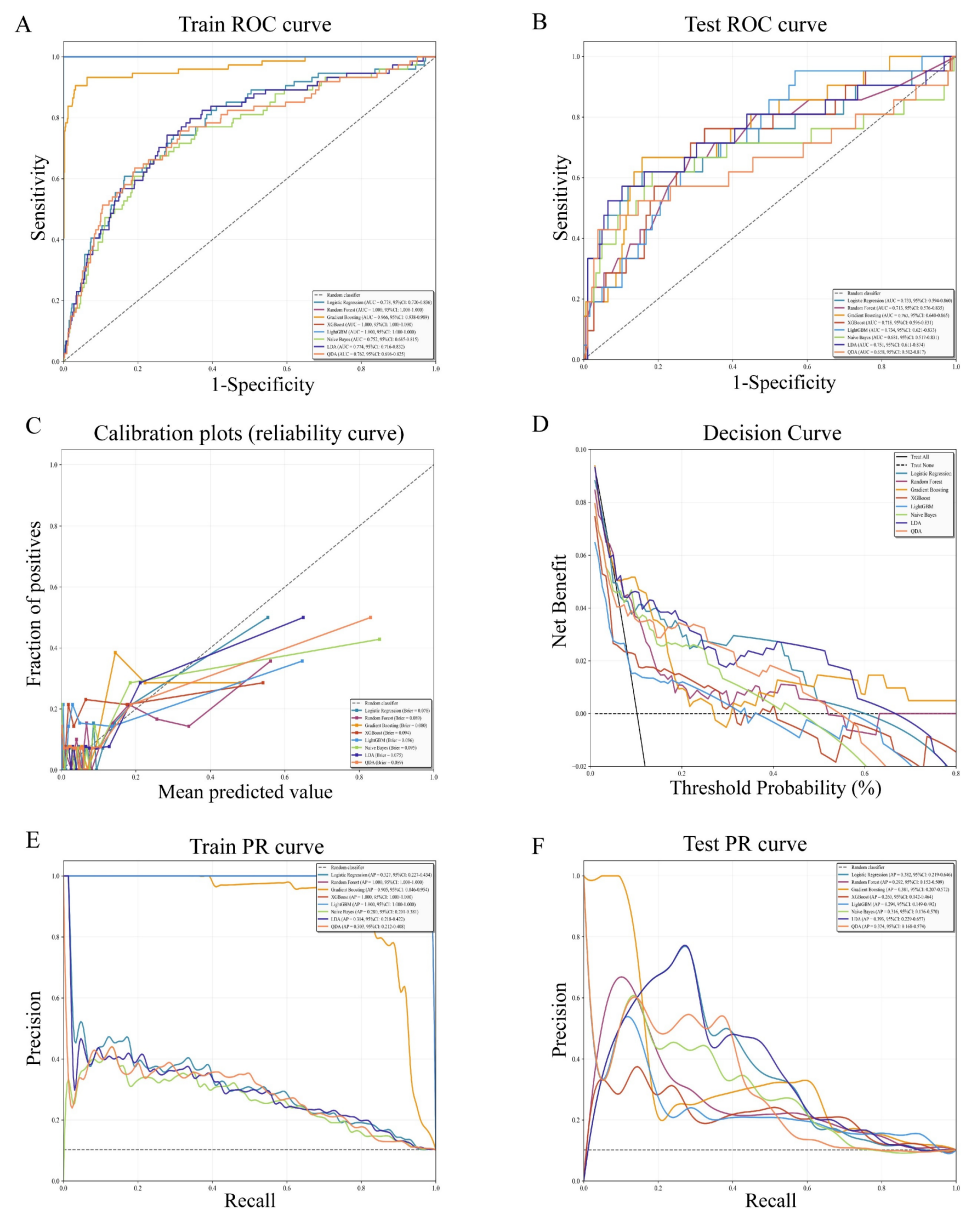
**Performance evaluation of eight machine learning models.** ROC curves in the training cohort (A) and test (B) cohort. (C) Calibration plots showing agreement between predicted probabilities and observed outcomes. (D) Decision curve analysis evaluating the clinical net benefit across a range of threshold probabilities. (E) Precision-recall (PR) curves in the training cohort. (F) PR curves in the test cohort. ROC: Receiver operating characteristic curve.

To enhance individualized prediction and clinical decision-making, eight machine learning models (Logistic Regression, Random Forest, Gradient Boosting, XGBoost, LightGBM, Naive Bayes, LDA, QDA) were developed and evaluated (Table 4 and Supplementary Figure 4). Results showed that Random Forest, XGBoost, LightGBM, and Gradient Boosting models achieved higher AUC values in the training set, while Gradient Boosting (AUC = 0.763, 95% CI: 0.640-0.865), LDA (AUC = 0.751, 95% CI: 0.611-0.874), and Logistic regression (AUC = 0.733, 95% CI: 0.594-0.860) maintained high AUC performance in the test set (Figure 3 A-B). In calibration plots, the Gradient Boosting, LDA, and Logistic regression model exhibited better calibration performance, as reflected by lower Brier scores (Brier score = 0.080, 0.075, and 0.078, respectively), indicating favorable consistency between predicted and observed risks (Figure 3C). Decision curve analysis showed that LDA, Logistic regression, and Gradient Boosting model achieved higher net benefit over a broader clinically relevant threshold probabilities compared with the treat-all (black solid line) and treat-none (black dashed line) strategies (Figure 3D). Precision-recall (PR) curve analysis was performed to further validate the reliability and clinical practicability of the predictive models (Figure 3E-F). In the training cohort, Gradient Boosting, Random Forest, LightGBM, and XGBoost model yielded a higher average precision, while in the test cohort, LDA (AP = 0.393, 95% CI: 0.229-0.657), Logistic regression (AP = 0.382, 95% CI: 0.219-0.646), and Gradient Boosting model (AP = 0.381, 95% CI: 0.207-0.572) showed higher AP and more stable precision at moderate recall levels. Collectively, considering discrimination, calibration, clinical utility, and robustness under class imbalance, the Gradient Boosting model emerged as the optimal model for predicting the risk of EAH/EC in asymptomatic postmenopausal women with endometrial thickening.

### 3.4 Assessment of the best-performing model

**Figure 4:**
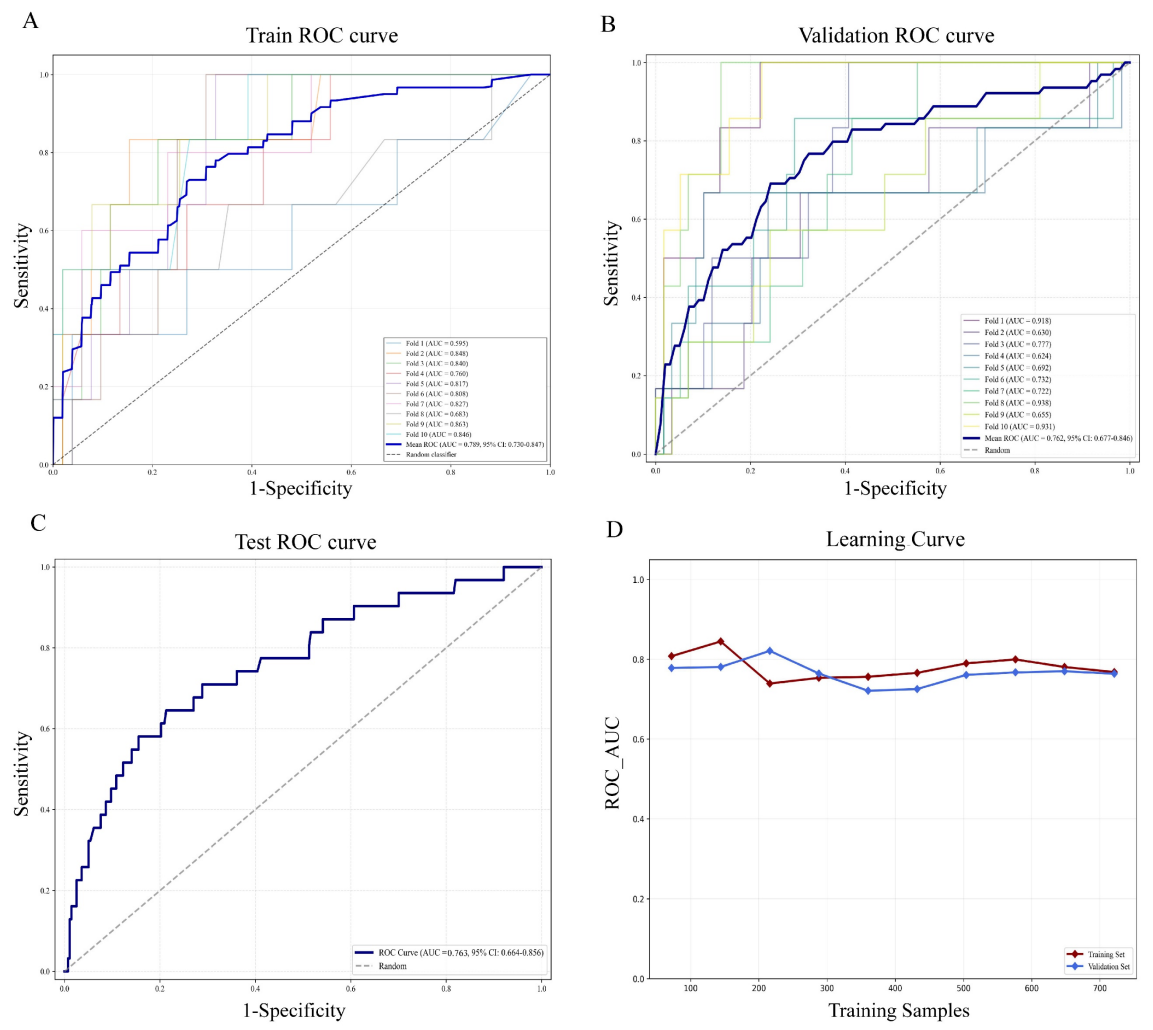
** Assessment of the gradient boosting model in training, validation, and testing sets.** (A) ROC curves and AUC values in the training cohort via ten-fold cross-validation. The blue bold line indicates the mean ROC curve, and each colored line represents an individual fold ROC curve. (B) ROC curves in the validation cohort. (C) ROC performance and AUC results for the independent test cohort (30% of malignant patients). (D) Learning curve showing changes in ROC AUC for the training (red solid line) and validation (blue solid line) sets with increasing training sample size. ROC, Receiver operating characteristic curve; AUC, Area under the ROC curve.

Gradient boosting analysis and 10-fold cross validation were carried out in the training set. As shown in Figure 4A-B, ROC curves for each fold demonstrated stable performance with mild variation, and the mean ROC curve differed markedly from the diagonal, suggesting favorable discriminative ability beyond random classification. The findings revealed that the average AUC of the training set was 0.789 (95% CI: 0.730-0.847) and the AUC of the validation set was 0.762 (95% CI: 0.677-0.846). In the test cohort, the model achieved an AUC of 0.763 (95% CI: 0.664-0.856) (Figure 4C). Learning curve analysis further supported the stability of the model, as AUC values for the training and validation sets converged with increasing sample size and remained closely aligned (Figure 4D). Taken together, the model maintained stable AUC values of around 0.76 in the training, validation, and test sets, demonstrating reliable predictive accuracy and satisfactory model fitting. These results demonstrated that the Gradient Boosting model was well-suited for the classification modeling task in this dataset.

### 3.5 Optimal model interpretability

**Figure 5:**
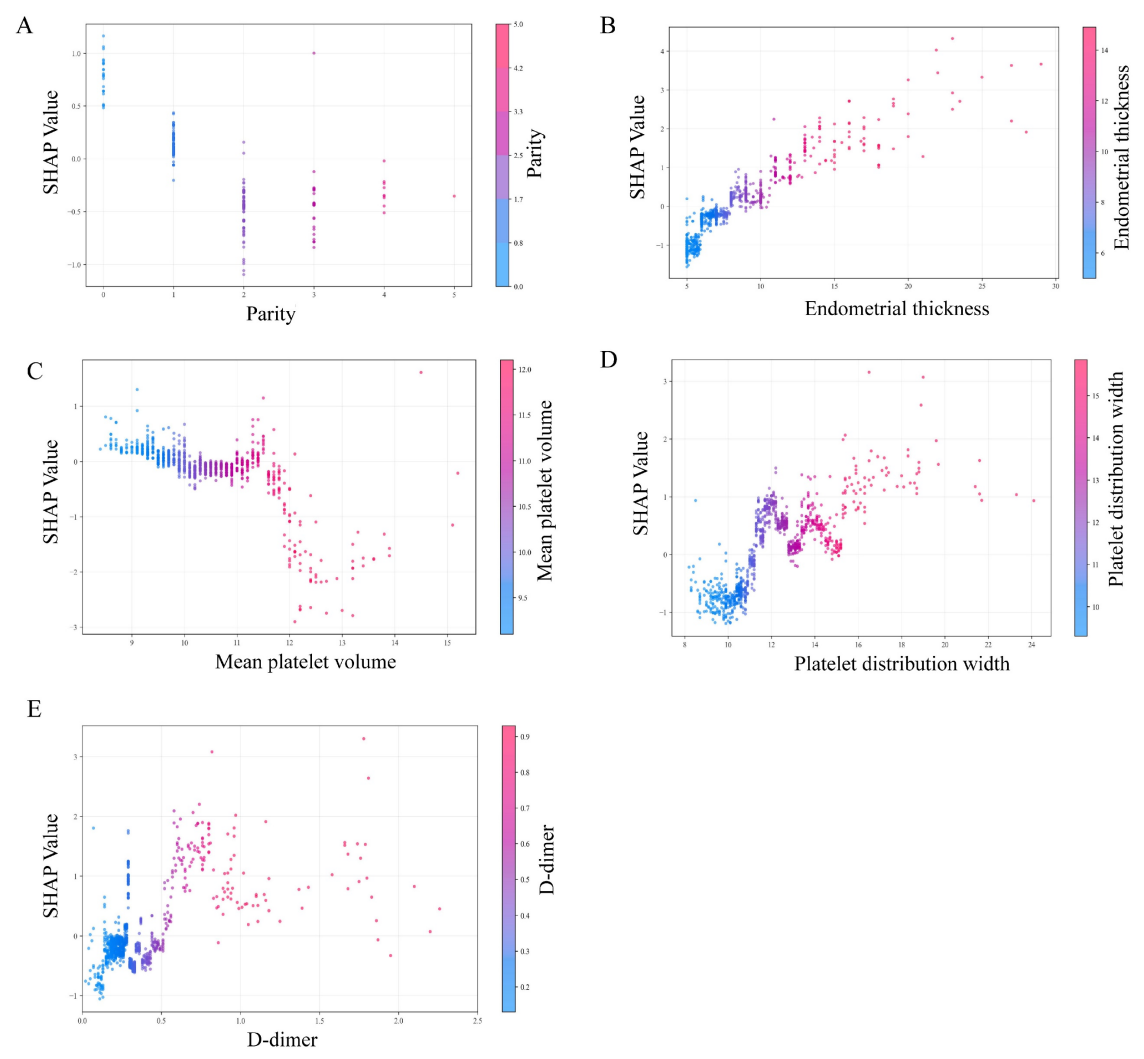
** SHAP dependence plots of model parameters.** (A) Parity, (B) endometrial thickness, (C) mean platelet volume (MPV), (D) platelet distribution width (PDW), and (E) D-dimer. Each dot represents an individual patient. The x-axis shows the feature value, and the y-axis indicates the corresponding SHAP value, reflecting the direction and magnitude of that feature's contribution to the predicted risk of endometrial non-benign lesions. Color gradients denote the feature value (low to high). SHAP, SHapley Additive exPlanations.

**Figure 6:**
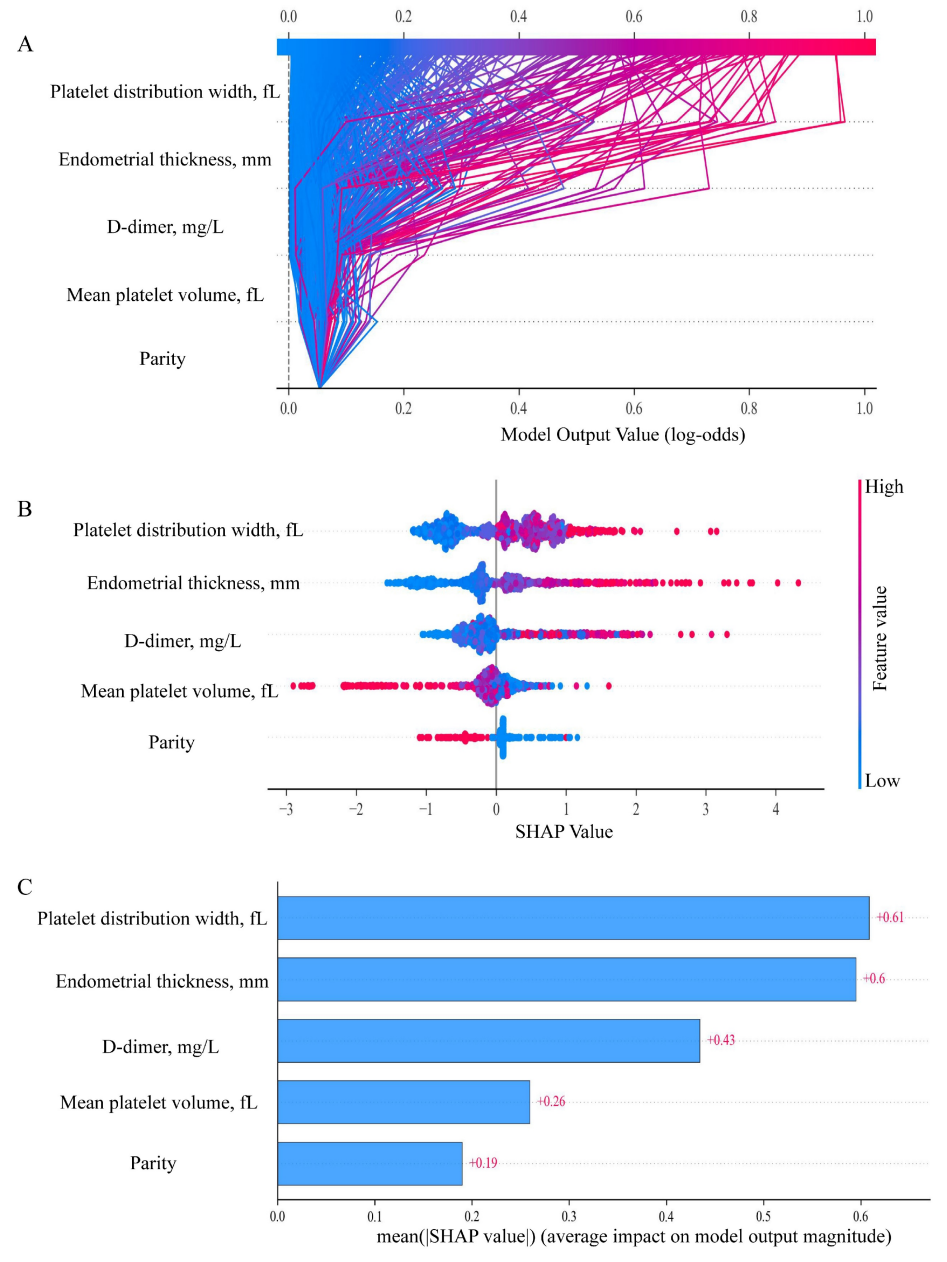
** SHAP-based global interpretation of the model.** (A) SHAP decision plot showing cumulative contributions of predictors to the model output (log-odds). Features are arranged on the y-axis according to their mean absolute SHAP values, with higher positions indicating a stronger contribution to the model's prediction. Each line represents an individual prediction trajectory: red indicates higher feature values that shift the model output toward an increased predicted risk of EAH/EC, whereas blue denotes lower feature values that move the prediction toward a lower or benign risk. (B) The SHAP summary plot. Each row represents one predictor, and each point corresponds to an individual patient. The x-axis shows the SHAP value, indicating whether the feature increases (positive values) or decreases (negative values) the predicted risk. Point color reflects the original feature value, with red indicating higher values and blue indicating lower values. (C) Feature importance ranked by mean absolute SHAP values. The plot illustrates the relative contribution of each covariate to the model predictions, with features ordered according to the magnitude of their overall impact. SHAP, SHapley Additive exPlanations.

To visually interpret the selected variables, we applied SHAP to illustrate how these variables contributed to the prediction of endometrial malignancy in the model. SHAP dependence plots for key variables were presented in Figure 5. We found that patients with elevated endometrial thickness, PDW, and D-dimer values (increase in the x-axis value) were associated with a higher SHAP value (increase in the y-axis value), which implied a greater probability of developing endometrial malignancy. Additionally, increased parity and MPV values were associated with lower SHAP values, indicating a lower likelihood of endometrial malignancy. Then, the decision plots shown in Figure 6A provided a comprehensive global overview of EAH/EC risk prediction.

In the SHAP summary plot (Figure 6B), each horizontal line represented one predictor, and each point corresponded to an individual patient. The x-axis showed the SHAP value, indicating whether the feature increased (positive values) or decreased (negative values) the predicted risk. Point color reflected the original feature value, with red indicating higher values and blue indicating lower values. Results illustrated that elevated PDW, thicker endometrial thickness, and higher D-dimer levels contributed to an increased risk of EAH/EC. Conversely, the associated risk was decreased by elevated parity and MPV. Moreover, Figure 6C demonstrated the importance ranking of five risk factors evaluated by the average absolute SHAP value, and PDW, endometrial thickness, and D-dimer were the top three important variables.

**Figure 7:**
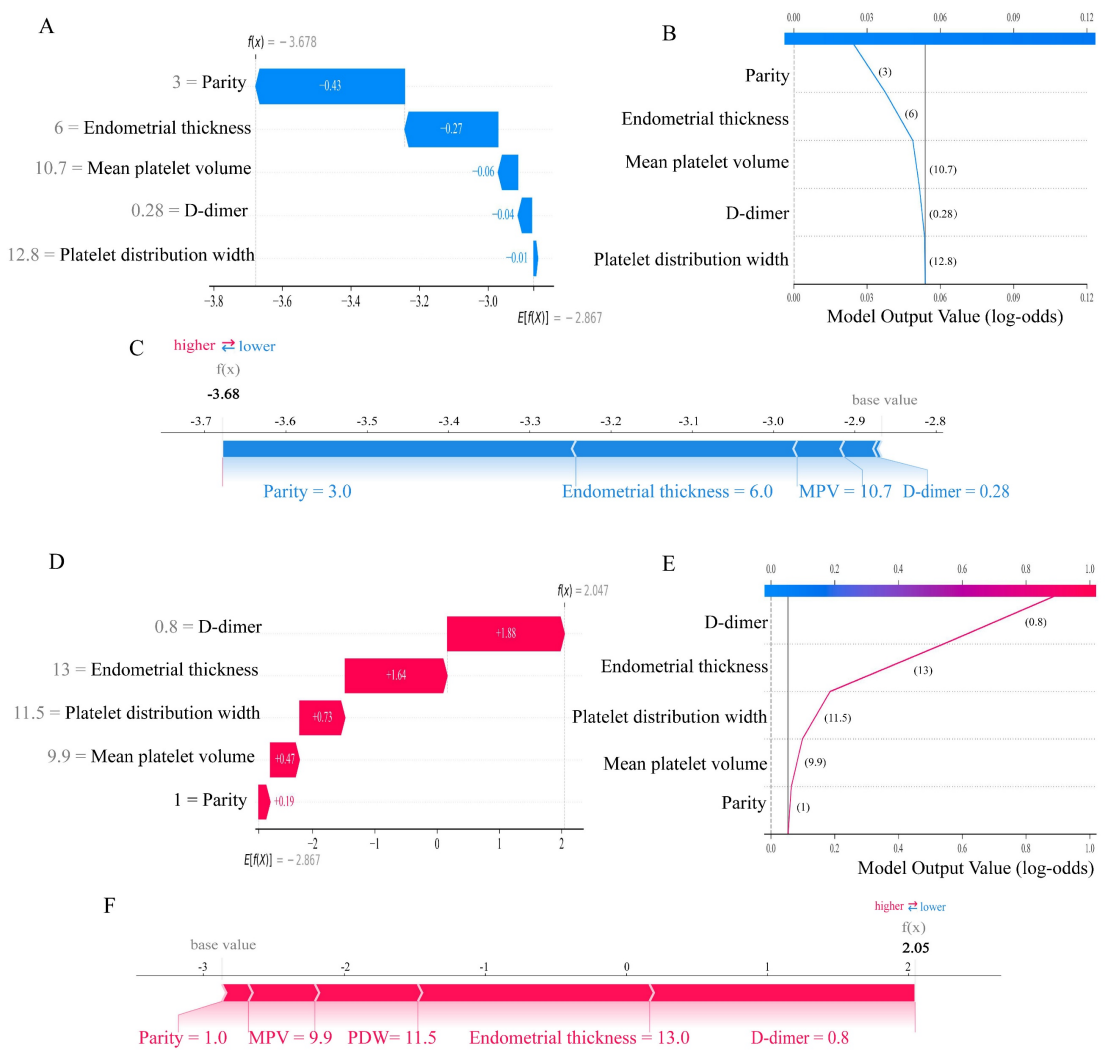
**Personalized interpretation of the model by SHAP.** SHAP waterfall plot (A), decision plot (B), and force plot (C) of patient 1, who was diagnosed with a benign lesion (true negative). SHAP waterfall plot (D), decision plot (E), and force plot (F) of patient 2, who was diagnosed with EC (true positive). In the waterfall plot, the red bar indicates the variable's contributory effect toward EAH/EC in the individual, while the blue bar represents its inhibitory effect, bar length corresponds to contribution magnitude. The f(X) values are the probabilistic predictions from the model, and E[f(X)] (the SHAP reference value) represents the model's predicted baseline in the absence of any input. The force plot illustrates how individual features contribute additively to the model's prediction by shifting the baseline decision score to the final predicted value. In all plots, the base value represents the average model output, and SHAP values indicate the direction and magnitude of each variable's contribution to the final prediction. Blue indicates lower risk contribution, and red indicates higher risk contribution. SHAP, SHapley Additive exPlanations. EAH/EC, Endometrial atypical hyperplasia/Endometrial carcinoma.

The SHAP method was advantageous in that it could not only enable global interpretability at the cohort level but also allowed for individualized interpretation of predictions at the patient level. To elucidate feature contributions for individual patients using the Gradient Boosting model, we employed two representative cases (Figure 7). One patient was diagnosed with endometrial polyp, belonging to the “true negative” subgroup, achieved a low probability of endometrial malignant lesions (Figure 7A-C), while another patient was pathologically diagnosed with EC, belonging to the “true positive” group, obtained an accurate prediction of high susceptibility to malignancy (Figures 7D-F).

## 4. Discussion

Clinical operations such as biopsy or hysteroscopy are commonly performed on asymptomatic postmenopausal individuals with endometrial thickening 29, yet most pathological results confirm benignity, resulting in avoidable surgery. Presently, there is a lack of optimal preoperative models that ensure timely cancer detection while avoiding overtreatment. This study innovatively developed and validated a machine learning-based predictive model for estimating the probability of endometrial malignancy, with parity, endometrial thickness, mean platelet volume (MPV), platelet distribution width (PDW), and D-dimer as core variables. Among eight ML models, the Gradient Boosting algorithm exhibited robust performance, characterized by excellent discriminative and calibration capabilities, and yielded notable net benefit in clinical practice. For a more in-depth understanding of the model, we applied the SHAP method for visualization and the model exhibited superior predictive efficacy. Moreover, to promote its clinical utility, we constructed an interactive web-based nomogram, empowering physicians to stratify patients timely and accurately before surgery, with the aim of avoiding redundant invasive interventions.

The management of incidentally asymptomatic endometrial thickening in postmenopausal women is a subject of debate 30. According to the American College of Obstetricians and Gynecologists (ACOG), women with postmenopausal uterine bleeding could be initially evaluated by endometrial biopsy or transvaginal ultrasonography, endometrial thickness above 4 to 5 mm by transvaginal ultrasound scan is identified as an indication for endometrial biopsy with or without hysteroscopy 31. Among postmenopausal women without abnormal bleeding, neither a standardized endometrial thickness threshold nor a validated predictive model for endometrial sampling currently exists. Our findings indicated that an optimal ET threshold of 8.15 mm for hysteroscopy was determined using the Youden index, with a maximum AUC of 0.751 (95% CI 0.693-0.808, p < 0.001), a sensitivity of 68%, and a specificity of 71%. However, the cutoff value determined by the optimal Youden index represented a statistically balanced threshold 32, 33, whereas clinicians may choose a more sensitive cutoff according to actual clinical demand to reduce false negatives and avoid missed diagnoses in different researches. In consistence with our results, Zhang, L. *et al.* revealed that an ET cut-off of 8 mm showed a reasonable performance to detect atypical hyperplasia (AH) and EC in this population 34. Schmidt *et al.*, however, showed 6 mm as a cut-off level for ET 35. Aggarwal, A. *et al.* advocated that a 10-mm cut for hysteroscopy was a safe practice 31. Additionally, Smith-Bindman *et al.* showed that endometrial biopsy should be considered when ET was greater than 11 mm 36. These wide variations could be attributed to variations in study frameworks, patient demographics, and enrolled patient numbers, particularly the proportion of premalignant and malignant cases included in each study. Given that our study encompassed a relatively considerable number of cases with premalignant or malignant lesions (n = 106), the obtained results exhibit robust and reliable validity.

In conjunction with the assessment of ET, we systematically synthesized demographic indicators (including comorbidities, such as hypertension, hyperlipidemia, and diabetes), inflammatory indices, and coagulation-associated factors to implement a comprehensive analysis. Through univariate, multivariate, and LASSO analysis, we innovatively identified five independent risk factors with potential predictive value for endometrial malignancy, encompassing ET, MPV, PDW, and D-dimer. Functional platelet abnormalities have been considered as part of paraneoplastic syndromes of many tumors 37. Platelet count (PLT), MPV, and PDW are three main indices utilized to evaluate platelet function and activation. In our study, no significant difference in PLT was observed between the benign and non-benign groups, whereas MPV and PDW were found to be significantly higher in non-benign patients than in their benign counterparts. Consistently, Yayla Abide, C. *et al.* showed that MPV was demonstrated to be significantly higher in EC patients compared with healthy individuals 38. Likewise, Chen, H. *et al.* also revealed that large platelets were released into the circulation during the early inflammatory phase of cancer, resulting in elevated PDW levels 39. Even so, their work did not target asymptomatic patients with endometrial thickening. As coagulation-related indicators in EC were less concerned, we applied them for the first time to predict cancer risk in asymptomatic patients with endometrial thickening and found that elevated D-dimer values were abnormally conspicuous in this asymptomatic population with endometrial malignancy. Similarly, Li, H. *et al.* detected that abnormal D-dimer values acted as a correlated risk factor associated with the incidence of EC, which was concordant with our results 40 When these independent risk factors were integrated into the nomogram, the AUC value was found to increase significantly to 0.784 (95%CI: 0.726-0.842), which was higher than that of any single marker, which demonstrated efficient discrimination for identifying the real high-risk subpopulation. Moreover, to enhance clinical usability, we developed a web calculator (https://yixuelietu.shinyapps.io/Dynamic_Nomogram/) to help clinicians easily and efficiently obtain the probability of EAH/EC in asymptomatic patients with endometrial thickening after menopause, thus assisting in determining whether invasive surgical intervention is warranted.

Compared to conventional statistical methods, ML can handle complex interactions by deciphering multidimensional predictor-outcome relationships 41. This study systematically evaluated and compared eight ML algorithms (Logistic Regression, Random Forest, Gradient Boosting, XGBoost, LightGBM, Naive Bayes, LDA, QDA) within a methodologically rigorous framework. Among them, the Gradient Boosting model consistently outperformed the others across all metrics, achieving an AUC of 0.966 in the training cohort and 0.763 in the internal validation cohort. During the analytical process, to avoid overfitting, we adopted several strategies, including rigorous feature selection via LASSO and model calibration evaluation. Decision curve analysis for validation cohort also confirmed its clinical applicability, implying that the Gradient Boosting-based model could be a valuable tool for early EC screening and risk stratification in asymptomatic postmenopausal women. In contrast, Stewart, A. *et al.*, like most other studies, merely reported a cut-off value for endometrial sampling in postmenopausal women without bleeding, they neither performed a comprehensive analysis combined with serological indicators nor established a functional predictive model 42, 43. To strengthen interpretability and clinical usability, we further leveraged SHAP analysis to quantify each feature's contribution to the model output, visualizing individualized and combined biomarker impacts on EC risk. This approach narrowed the gap between model accuracy and clinical transparency, allowing clinicians to better understand the biological basis of predictions and make evidence-based decisions. It could also promote personalized screening strategies-especially in high-incidence populations-to enhance early diagnosis and patient outcomes.

The present study not only strengthened methodological robustness through temporal validation but, crucially, integrated machine learning algorithms and SHAP framework via utilizing innovative serological and ultrasound metrics, thereby enhancing both model efficacy and clinical applicability while improving interpretability. The main strengths of the present investigation lay in the large sample size of EAH/EC patients. In fact, to the best of our knowledge, no other online dynamic nomogram has been developed to improve the precision of clinical triage for malignancy in asymptomatic postmenopausal women. Moreover, the predictors were derived from regular clinical tests, which were objective, inexpensive and readily obtainable. Clinicians could use this interactive visualization tool to identify postmenopausal women at high risk of EAH/EC, potentially reducing the need for invasive procedures such as hysteroscopy in low-risk individuals.

While the findings were encouraging, several limitations should be acknowledged. First, some potential biases, such as selection bias, missing data biases, might compromise the reliability of the findings. Second, although the utilization of internal cross-validation method was performed to mitigate the risk of overfitting, the absence of external validation cohort would restrict assessment of the model's reproducibility to some degree. Future multicenter studies across diverse geographic regions and heterogeneous populations were essential to evaluate its generalizability. Lastly, additional biomarkers, including radiomics data as well as transcriptomic and proteomic biomarkers, might have the capacity to boost prediction precision. Future investigations should explore the integration of these factors to further refine the Gradient Boosting model described in this research.

## 5. Conclusions

In this large-scale study, we characterized a sensitive array of clinicopathologic, serological, and ultrasonic indicators-parity, ET, MPV, PDW, and D-dimer associated with EAH/EC risk. Based on these easily attainable predictors, a dynamic online nomogram was built, which provided a practical tool for risk assessment. Furthermore, we constructed and validated a Gradient Boosting-based predictive model that achieved outstanding performance across internal training and validation cohorts. Meanwhile, SHAP-based interpretation enabled a scalable strategy for early risk stratification of asymptomatic postmenopausal women with endometrial thickening, thereby assisting clinicians in optimizing hysteroscopy intervention decisions via an efficient computer-aided approach.

## Supplementary Material

Supplementary figures and table.

## Figures and Tables

**Table 1:** Pathological characteristics in postmenopausal patients with thickened endometrium.

Pathology result (Total n = 1031)	n	%
Benign discoveries	925	89.7
Endometrial polyps	657	63.7
Simple hyperplasia	143	13.8
Complex hyperplasia	34	3.3
Leiomyoma	27	2.6
Endometritis	18	1.7
Atypical endometrial hyperplasia	30	2.9
Endometrial carcinoma	76	7.4

**Table 2:** Characteristics of the training cohort stratified by pathological diagnosis.

Characteristics	Overall (n = 774)	Benign lesions (n = 693)	EAH/EC (n = 81)	P value
Age, yr	63 (58, 68)	64 (58, 68)	61 (55, 66.5)	0.017
Duration of menopause, yr	10 (6, 16)	10 (6, 16)	10 (3.7, 15)	0.029
Age at menopause, yr	51 (50, 53)	51 (49.7, 53.5)	51 (49.7, 53)	0.558
Hypertension				0.361
Yes	333 (43.0)	302 (43.6)	31 (38.3)	
No	441 (57.0)	391 (56.4)	50 (61.7)	
Diabetes				0.681
Yes	87 (11.2)	79 (11.4)	8 (9.9)	
No	687 (88.8)	614 (88.6)	73 (90.1)	
Hyperlipidemia				0.326
Yes	42 (5.4)	40 (5.8)	2 (2.5)	
No	732 (94.6)	653 (94.2)	79 (97.5)	
Gravidity				0.428
0	15 (1.9)	12 (1.7)	3 (3.7)	
≥ 1	759 (98.1)	681 (98.3)	78 (96.3)	
Parity				0.043
0	24 (3.1)	18 (2.6)	6 (7.4)	
≥ 1	750 (96.9)	675 (97.4)	75 (92.6)	
Miscarriages	1.0 (1.0, 2.0)	1.0 (0.0, 2.0)	1.0 (0.0, 2.0)	0.595
BMI, kg/m^2^	24.2 (22.3,26.6)	24.1 (22.3,26.6)	24.8 (22.2,27.0)	0.356
Endometrial thickness, mm	8.14±3.33 7 (6, 9)	7.77±2.89	11.37±5.11	< 0.001
PLT, ×10^9^/L	224 (193, 263)	224 (193, 262)	227 (202.5,264)	0.425
WBC, ×10^9^/L	6 (5, 7)	6 (5, 7)	6.2 (5.2, 7.4)	0.376
Neutrophils, ×10^9^/L	3.5 (2.9, 4.5)	3.56 (2.86, 4.46)	3.78 (3.03, 4.69)	0.136
Lymphocytes, ×10^9^/L	1.7 (1.4, 2.1)	1.75 (1.39, 2.13)	1.71 (1.35, 2.07)	0.809
Monocytes, ×10^9^/L	0.38 (0.31, 0.48)	0.38 (0.31, 0.48)	0.41 (0.30, 0.49)	0.623
HCT, %	39.9 (38.2, 42.3)	39.9 (38.2, 42.3)	40.5 (37.5, 42.1)	0.924
MCV, fL	91.3 (88.8, 93.8)	91.2 (88.8, 93.9)	92.0 (88.8, 93.6)	0.871
RDW, %	12.5 (12.1, 12.9)	12.5 (12.1, 12.9)	12.6 (12.2, 13.1)	0.007
MPV, fL	10.3 (9.7, 11.0)	10.3 (9.7, 10.9)	10.6 (10.0, 11.2)	0.024
PDW, FL	11.7 (10.6, 13.2)	11.7 (10.6, 13.1)	12.5 (11.4, 13.8)	< 0.001
P-LCR, %	27.5 (22.6, 32.8)	27.3 (22.4, 32.7)	29.1 (25.3, 34.5)	0.010
PCT, %	0.23 (0.21, 0.27)	0.23 (0.20, 0.27)	0.24 (0.21, 0.28)	0.260
PT, s	12.5 (12.1, 12.9)	12.5 (12.1, 12.9)	12.6 (12.2, 13.0)	0.318
INR	0.96 (0.92, 0.99)	0.95 (0.92, 0.99)	0.96 (0.93, 1.00)	0.265
APTT, s	34.3 (31.7, 36.5)	34.3 (31.6, 36.6)	34.7 (32.5, 36.3)	0.388
Fibrinogen, g/L	3.05 (2.75, 3.37)	3.07 (2.75, 3.37)	3.05 (2.80, 3.39)	0.544
TT, s	16.9 (16.3, 17.6)	16.9 (16.3, 17.6)	17.0 (16.3, 17.9)	0.257
D-dimer, mg/L	0.28 (0.20, 0.39)	0.27 (0.20, 0.38)	0.29 (0.24, 0.65)	< 0.001
FDP, mg/L	1.62 (1.15, 1.97)	1.62 (1.15, 1.97)	1.62 (1.06, 1.88)	0.877
NLR	2.09 (1.57, 2.65)	2.05 (1.55, 2.62)	2.27 (1.70, 2.71)	0.239
PLR	130.3 (103.6,162.9)	130.1 (103.2, 163.9)	131.7 (108.0,153.1)	0.733
MLR	0.22 (0.18, 0.28)	0.21 (0.18, 0.27)	0.23 (0.17, 0.27)	0.797

**Abbreviations:** EAH, endometrial atypical hyperplasia; EC, endometrial carcinoma; BMI, body mass index; PLT, platelet count; WBC, white blood cells; HCT, hematocrit; MCV, mean corpuscular volume; RDW, red cell distribution width; MPV, mean platelet volume; PDW, platelet distribution width; P-LCR, platelet-larger cell ratio; PCT, platelet thrombocytocrit; NLR, neutrophil-to-lymphocyte ratio; PLR, platelet-to-lymphocyte ratio; MLR, monocyte-to-lymphocyte ratio; PT, prothrombin time; INR, international normalized ratio; APTT, activated partial thromboplastin time; TT, thrombin time; FDP, fibrin degradation products.

**Table 3:** Multivariable analysis of the training cohort.

	Multivariate analysis			Selected factors for model	
Risk factors	OR (95% CI)	P value		OR (95% CI)	P value
Age, yr	0.939 (0.873-1.010)	0.092			
Duration of menopause, yr	1.011 (0.947-1.080)	0.736			
Gravidity	1.038 (0.825-1.306)	0.749			
Parity	0.553 (0.307-0.994)	0.048		0.522 (0.314-0.868)	0.012
BMI, kg/m^2^	1.007 (0.938-1.082)	0.842			
Endometrial thickness, mm	1.252 (1.179-1.330)	0.000		1.250 (1.178-1.327)	< 0.001
RDW, %	0.961 (0.837-1.102)	0.567			
MPV, fL	0.191 (0.049-0.755)	0.018		0.387 (0.165-0.913)	0.030
PDW, FL	1.650 (1.098-2.479)	0.016		1.636 (1.130-2.368)	0.009
P-LCR, %	1.092 (0.930-1.283)	0.283			
D-dimer, mg/L	1.591 (1.161-2.180)	0.004		1.538 (1.130-2.094)	0.006

**Abbreviations:** BMI, body mass index; RDW, red cell distribution width; MPV, mean platelet volume; PDW, platelet distribution width; P-LCR, platelet-larger cell ratio; OR, odds ratio; CI, confidence interval.

**Table 4:** Performance of machine learning models in the training and test datasets.

Cohort	Model	AUC	AUC 95% CI	Acc	Acc 95% CI	Sen	Spe	PPV	NPV
Train	Gradient Boosting	0.966	0.938-0.989	0.839	0.814-0.863	0.932	0.828	0.383	0.991
Test	Gradient Boosting	0.763	0.640-0.865	0.791	0.733-0.845	0.667	0.805	0.280	0.955
Train	LDA	0.774	0.716-0.832	0.821	0.793-0.849	0.527	0.855	0.293	0.940
Test	LDA	0.751	0.611-0.874	0.864	0.816-0.908	0.571	0.897	0.387	0.949
Train	LightGBM	1.000	1.000-1.000	0.946	0.929-0.963	1.000	0.940	0.655	1.000
Test	LightGBM	0.734	0.621-0.833	0.811	0.752-0.859	0.333	0.865	0.219	0.920
Train	Logistic Regression	0.778	0.720-0.836	0.897	0.875-0.920	0.054	0.994	0.500	0.902
Test	Logistic Regression	0.733	0.594-0.860	0.903	0.859-0.942	0.143	0.989	0.600	0.910
Train	Naive Bayes	0.752	0.685-0.815	0.752	0.721-0.784	0.649	0.764	0.239	0.950
Test	Naive Bayes	0.681	0.517-0.831	0.796	0.743-0.845	0.619	0.816	0.277	0.950
Train	QDA	0.762	0.696-0.825	0.867	0.842-0.893	0.365	0.924	0.355	0.927
Test	QDA	0.658	0.502-0.817	0.908	0.869-0.947	0.429	0.962	0.563	0.937
Train	Random Forest	1.000	1.000-1.000	0.970	0.957-0.981	1.000	0.966	0.771	1.000
Test	Random Forest	0.712	0.576-0.835	0.806	0.752-0.854	0.429	0.849	0.243	0.929
Train	XGBoost	1.000	1.000-1.000	0.985	0.974-0.993	1.000	0.983	0.871	1.000
Test	XGBoost	0.718	0.596-0.831	0.850	0.796-0.893	0.286	0.914	0.273	0.918

**Abbreviations:** AUC, area under the receiver operating characteristic curve; Acc, accuracy; Sen, sensitivity; Spe, specificity; PPV, positive predictive value; NPV, negative predictive value; LDA, linear discriminant analysis; QDA, quadratic discriminant analysis; LightGBM, Light Gradient Boosting Machine; XGBoost, extreme gradient boosting; CI, confidence intervals.

## Data Availability

The data that support the findings of this study are available from the corresponding author upon reasonable request.

## Abbreviations

ET: endometrial thickness
EC: endometrial carcinoma
EAH: endometrial atypical hyperplasia
SHAP: SHapley Additive exPlanations
LASSO: Least Absolute Shrinkage and Selection Operator
ML: machine learning
ROC: Receiver Operating Characteristic
AUC: area under the ROC curve
LR: logistic regression
RF: random forest
XGBoost: extreme gradient boosting
LDA: linear discriminant analysis
QDA: quadratic discriminant analysis
DCA: decision curve analysis
PR: precision-recall
AUPRC: the area under the PR curve
BMI: body mass index
WBC: white blood cells
PLT: platelet count
HCT: hematocrit
MCV: mean corpuscular volume
RDW: red cell distribution width
MPV: mean platelet volume
PDW: platelet distribution width
P-LCR: platelet-larger cell ratio
PCT: platelet thrombocytocrit
PT: prothrombin time
INR: international normalized ratio
APTT: activated partial thromboplastin time
TT: thrombin time
FDP: fibrin degradation products
NLR: neutrophil-to-lymphocyte ratio
PLR: platelet-to-lymphocyte ratio
MLR: monocyte-to-lymphocyte ratio
