# Supplementary material for: Early Identification of Endometrial Malignancy in Postmenopausal Women with Asymptomatic Endometrial Thickening: A Novel Explainable Machine Learning Model
Source: Int J Med Sci. 2026 Mar 25;23(5):1667–84. doi: 10.7150/ijms.131192 (PMC13133880; doi:10.7150/ijms.131192)

Supplementary Table 1 Baseline characteristics of the training and validation cohorts

Characteristics	Train (n = 774)	Valid (n = 257)	P value
Age, yr	63 (58, 68)	63 (58, 68)	0.395
Duration of menopause, yr	10 (6, 16)	10 (6, 17)	0.787
Age at menopause, yr	51 (50, 53)	52 (49, 54)	0.409
Hypertension			0.708
Yes	333 (43.0)	114 (44.4)	
No	441 (57.0)	143 (55.6)	
Diabetes			0.226
Yes	87 (11.2)	22 (8.6)	
No	687 (88.8)	235 (91.4)	
Hyperlipidemia			0.330
Yes	42 (5.4)	10 (3.9)	
No	732 (94.6)	247 (96.1)	
Gravidity			0.326
0	15 (1.9)	2 (0.8)	
≥ 1	759 (98.1)	255 (99.2)	
Parity			0.093
0	24 (3.1)	3 (1.1)	
≥ 1	750 (96.9)	254 (98.9)	
Miscarriages	1.0 (1.0, 2.0)	1.0 (1.0, 1.0)	0.390
BMI, kg/m^2	24.2 (22.3, 26.6)	24.2 (22.2, 26.6)	0.645
Endometrial thickness, mm	8.14±3.33	8.15 ±3.10	0.538
	7 (6, 9)		
PLT, ×10^9/L	224 (193, 263)	231 (197, 269)	0.195
WBC, ×10^9/L	6.0 (5.0, 7.0)	5.9 (5.1, 7.1)	0.733
Neutrophils, ×10^9/L	3.5 (2.9, 4.5)	3.5 (2.8, 4.4)	0.852
Lymphocytes, ×10^9/L	1.7 (1.4, 2.1)	1.8 (1.4, 2.2)	0.444
Monocytes, ×10^9/L	0.38 (0.31, 0.48)	0.38 (0.31, 0.49)	0.967
HCT, %	39.9 (38.2, 42.3)	39.9 (38.3, 41.9)	0.502
MCV, fL	91.3 (88.8, 93.8)	91.4 (88.7, 93.6)	0.738
RDW, %	12.5 (12.1, 12.9)	12.5 (12.2, 12.9)	0.893
MPV, fL	10.3 (9.7, 11.0)	10.3 (9.7, 11.0)	0.801
PDW, FL	11.7 (10.6, 13.2)	11.7 (10.4, 13.1)	0.715
P-LCR, %	27.5 (22.6, 32.8)	27.5 (22.5, 32.9)	0.999
PCT, %	0.23 (0.21, 0.27)	0.24 (0.21, 0.27)	0.761
PT, s	12.5 (12.1, 12.9)	12.5 (12.1, 13.0)	0.656
INR	0.96 (0.92, 0.99)	0.95 (0.92, 1.00)	0.390
APTT, s	34.3 (31.7, 36.5)	34.3 (32.0, 37.4)	0.429
Fibrinogen, g/L	3.05 (2.75, 3.37)	3.04 (2.68, 3.33)	0.397
TT, s	16.9 (16.3, 17.6)	16.9 (16.4, 17.6)	0.952
D-dimer, mg/L	0.28 (0.20, 0.39)	0.27 (0.21, 0.38)	0.675
FDP, mg/L	1.62 (1.15, 1.97)	1.60 (1.18, 2.04)	0.562

NLR	2.09 (1.57, 2.65)	2.06 (1.45, 2.67)	0.564
PLR	130.3 (103.6, 162.9)	129.2 (102.5, 162.8)	0.703
MLR	0.22 (0.18, 0.28)	0.22 (0.16, 0.28)	0.285

Data are presented as mean ± standard deviation for normally distributed continuous variables, median (interquartile range) for non-normally distributed continuous variables, and number (percentage) for categorical variables. Abbreviations: BMI, body mass index; PLT, platelet count; WBC, white blood cells; HCT, hematocrit; MCV, mean corpuscular volume; RDW, red cell distribution width; MPV, mean platelet volume; PDW, platelet distribution width; P-LCR, platelet-larger cell ratio; PCT, platelet thrombocytocrit; NLR, neutrophil count and lymphocyte count ratio; PLR, platelet count and lymphocyte count ratio; MLR, monocyte count and lymphocyte count ratio; PT, prothrombin time; INR, international normalized ratio; APTT, activated partial thromboplastin time; TT, thrombin time; FDP, fibrin degradation products.

Supplementary Figure 1. Histological subtypes of endometrial carcinoma

The horizontal bars represent the number of cases for each subtype, with percentages calculated relative to the total cohort.

Supplementary Figure 2. LASSO regression coefficients

LASSO regression coefficients of candidate predictors at the optimal penalty parameter (λ) selected via cross-validation. Positive coefficients indicate variables associated with increased risk of endometrial malignancy, whereas negative coefficients suggest a potential protective association. LASSO, least absolute shrinkage and selection operator; WBC, white blood cells; MPV, mean platelet volume; PDW, platelet distribution width; PCT, platelet thrombocytocrit; INR, international normalized ratio; TT, thrombin time.

Supplementary Figure 3. Performance evaluation of nomogram model

ROC curves evaluating the model's discrimination ability in the entire cohort (A), training cohort (B), and validation cohort (C). The dashed line represents the "ideal" (AUC=1.0) model; Calibration plots comparing the predicted probability (x-axis) and actual probability (y-axis) of the outcome in the entire (D), training (E), and validation (F) cohorts. The solid line denotes the model's performance; the dashed line is the "ideal" (perfect calibration) reference. DCA Curves assessing the clinical utility of the nomogram in the entire (G), training (H), and validation (I) cohorts. The y-axis represents standardized net benefit; the x-axis denotes the high-risk threshold (or cost:benefit ratio). ROC, Receiver operating characteristic curve. DCA, Decision curve analysis.

Supplementary Figure 4. Comparison of predictive performance across eight machine learning models in the training cohort.

(A) Area under the receiver operating characteristic curve (AUC), (B) accuracy, (C) sensitivity, (D) specificity, (E) positive predictive value (PPV), and (F) negative predictive value (NPV) of eight machine learning models. GB, Gradient Boosting; LDA, Linear Discriminant Analysis; LGBM, Light Gradient Boosting Machine; LR, Logistic Regression; XGB, Extreme Gradient Boosting; RF, Random Forest; NB, Naive Bayes; QDA, Quadratic Discriminant Analysis.

Supplementary Figure 1

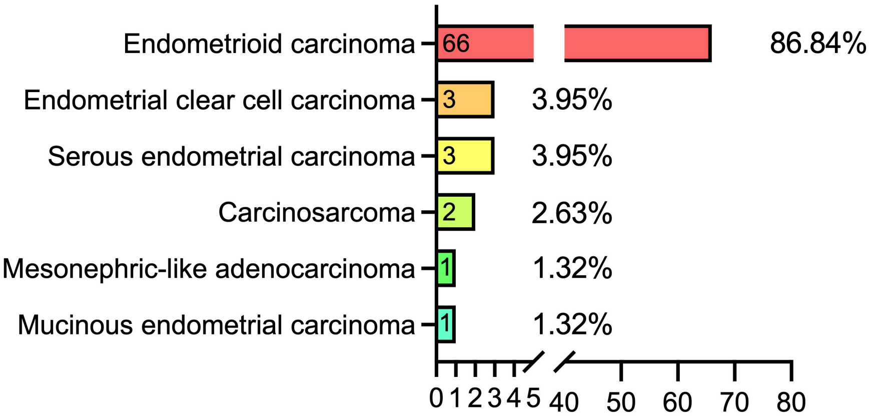

Histological subtypes of endometrial carcinoma（number of patients/percent）

Supplementary Figure 2

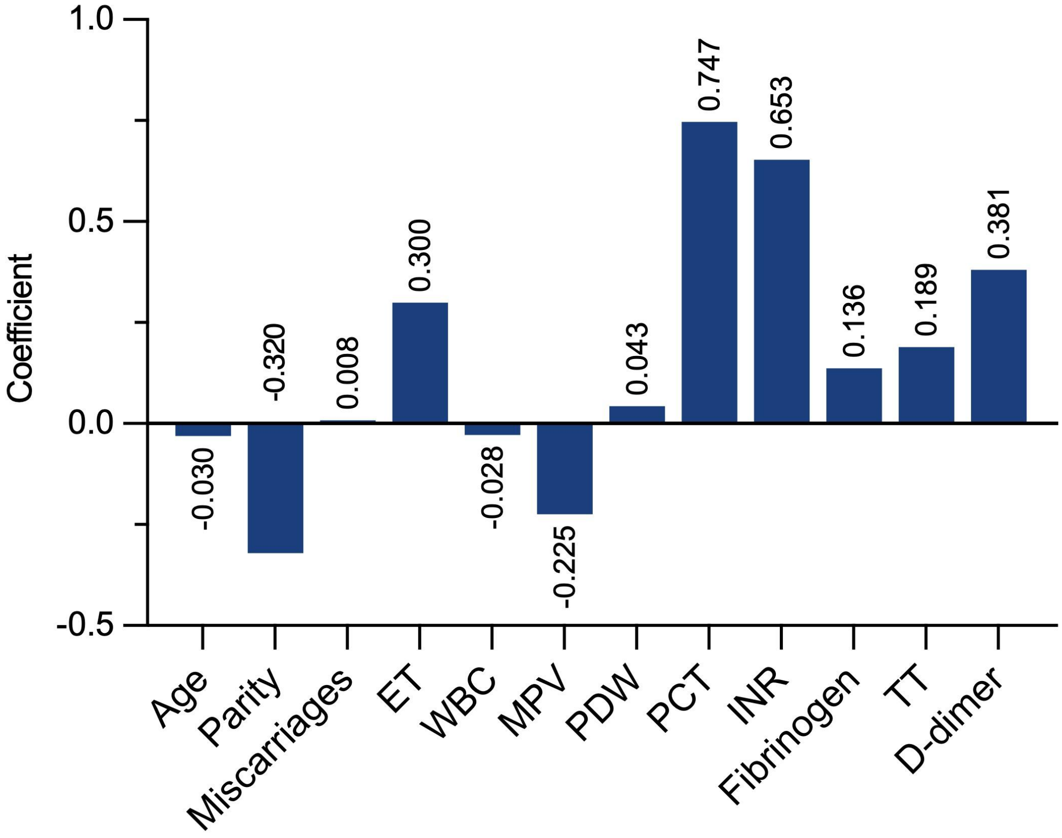

Supplementary Figure3

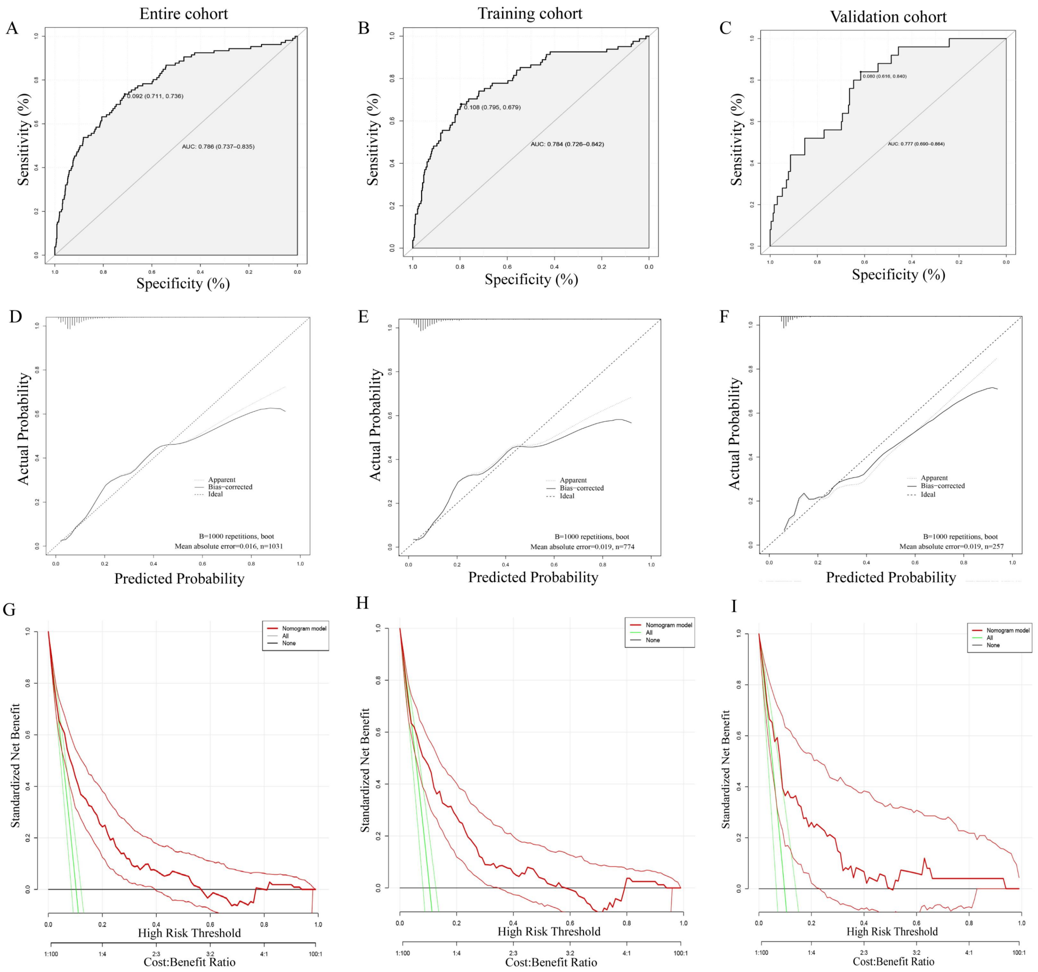

Supplementary Figure4

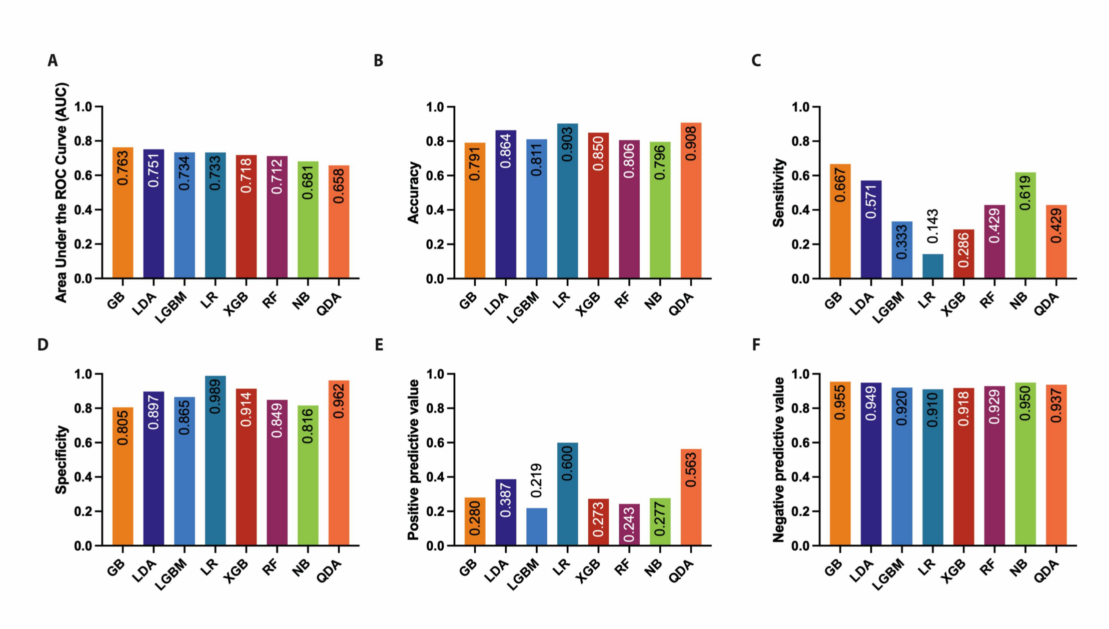

Supplement: Supplementary file 1 — Supplementary figures and table. [file ijmsv23p1667s1.pdf]
